# Comparison of growth in neutered Domestic Shorthair kittens with growth in sexually-intact cats

**DOI:** 10.1371/journal.pone.0283016

**Published:** 2023-03-15

**Authors:** Carina Salt, Richard F. Butterwick, Kristin S. Henzel, Alexander J. German

**Affiliations:** 1 Waltham Petcare Science Institute, Waltham on the Wolds, Leicestershire, United Kingdom; 2 Royal Canin Research Center, Aimargues, France; 3 Institute of Life Course and Medical Sciences, University of Liverpool, Neston, Cheshire, United Kingdom; Aristotle University of Thessaloniki School of Veterinary Medicine, GREECE

## Abstract

The first aim of these studies was to compare growth patterns of healthy kittens neutered during growth with growth standards created for sexually-intact kittens. A second aim was to clarify the impact of neutering in kittens on body composition and body shape. Study 1 was a retrospective observational study comparing median growth trajectories of healthy, client-owned domestic shorthair (DSH) kittens in optimal body condition and neutered at different ages, with previously-created growth standards from a similar, sexually-intact, population. The neuter groups contained between 3.0k and 9.3k cats. For all neuter groups in both sexes, the median growth trajectory inclined upwards after the procedure, with this being more marked in female than in male kittens. This upwards inclination was less marked for kittens neutered later during growth in both sexes, with the effect being least in kittens neutered after 28–29 weeks. Study 2 was an analysis of new body composition and zoometric data from a previously-published randomised study, comparing growth-related measures between 11 pairs of sexually-intact and neutered (at 19 weeks age) female DSH cats in a research population. Before neutering, the growth pattern in neutered kittens and sexually-intact kittens was similar, but neutered kittens were heavier by 52 weeks (mean difference in fold change vs. 10 weeks 1.34, 95-CI: 1.07–1.72), had a greater fat mass (mean difference in fold change vs. 10 weeks 1.91, 95-CI 1.09–3.21) and greater lean mass (mean difference in fold change vs. 10 weeks 1.23, 95-CI: 1.03–1.48). Abdominal girth (mean difference in fold change vs. 10 weeks 1.20, 95-CI: 1.04–1.39) and rib cage length (mean difference in fold change vs. 10 weeks 1.18, 95-CI: 1.02–1.36) were also greater, but there were no differences in other zoometric measurements. Veterinarians should consider the potential impact that neutering has on gain of adipose tissue, especially early neutering in female kittens. Bodyweight should be monitored closely during growth and especially after neutering to prevent inappropriate weight gain.

## Introduction

The owners of most pet cats in the developed world choose to have them neutered by surgical gonadectomy (castration or ovariohysterectomy for male and female cats, respectively) [1], which has perceived benefits including controlling the stray and pet cat populations, reducing undesirable behaviour (especially in male cats), and decreasing the risk of neoplasia of both the mammary gland and urogenital tract [2,3]. However, neutering can have adverse effects on health in cats including increased risk of feline lower urinary tract disease and increased risk of diabetes mellitus [4,5], as well as being a known risk factor for the development of obesity [6–9] characterised by an expansion of white adipose tissue mass that can lead to adverse health effects [10]. This obesity risk is thought to be associated with increased food intake, which peaks at 8–10 weeks after neutering, but declines to the intake seen in sexually-intact kittens by 18 weeks after neutering [8,9]. The effect is that neutered kittens fed *ad libitum* grow significantly faster and end their growth phase heavier than sexually-intact cats, both in terms of bodyweight and fat mass [8,9]. As a result, it is recommended that food intake be controlled after neutering to prevent the development of obesity [7]. Given the known effects of neutering on food intake and weight gain in cats, their impact on patterns of growth is pertinent, not least because most cats are neutered before 1 year of age [11,12].

Optimal growth is key priority for all species, especially those that act as companion animals including cats. Providing complete and balanced nutrition is important, but it is also important to ensure that the correct amount be fed so that the pattern of growth is optimal. Malnutrition at this critical stage can cause growth retardation [13,14], but this is uncommon. In reality, overnutrition is a greater concern, leading to overly rapid growth and increasing the risk of developing obesity [14–16]. Recently, evidence-based growth standards have been developed for male and female domestic shorthair cats [16] and show promise for use as a tool for monitoring the growth, not least given that body condition scoring [17] has not been validated for assessing body composition in kittens. The recently developed growth charts utilised data from cats that remained sexually-intact during growth, but the impact of neutering on such patterns was not studied [16]. In dogs, neutering had only a minor impact on growth trajectory, within one centile of the median line [18] and, as a consequence, standards for sexually-intact animals were appropriate for neutered animals. That said, the impact of neutering differed depending upon its timing [14]: a slight upward shift in growth trajectory was observed when neutering occurred before 37 weeks, whilst a slight downward shift in growth trajectory was observed in dogs neutered after 37 weeks. Therefore, in determining the effect of neutering on the pattern of growth in cats, the impact of timing is also likely to be important, and this is of interest because of recent shifts towards early-age neutering, with a study from Australia demonstrating 60% of cats being neutered before 6 months’ age and 22% before 4 months’ age [19]. Early-age neutering performed before puberty (5–9 month’s age) can effectively prevent unwanted litters in cats, thereby helping to control both stray and pet cat populations [20]. However, the physiological impact of neutering at such a stage of immaturity has not been explored in detail. In ome previous study, pre-pubertal neutering was not identified as a risk factor for overweight or obesity, compared with obesity at a traditional age (7 months) [20–22]. However, in a second study, early-age neutering resulted in a marked increase in food intake, increased bodyweight and also body fat mass, with the effects being most notable in female, compared with male, kittens [9].

The aim of the study was to inform recommendations around using the growth standards in neutered kittens. Given previous findings in dogs [16], we hypothesised that the growth pattern of neutered cats would not differ from that of sexually-intact kittens. Therefore, we investigated the growth patterns of healthy kittens of normal body condition score, neutered during growth, with those of the growth standards. Given that any increase in bodyweight could be due to a change in body composition or body size, we also explored the impact of neutering on body shape and body composition, by analysing new data gathered from a previous study assessing the effects of neutering on food intake, bodyweight and body composition in growing female kittens [9].

## Materials and methods

### Study design

This paper reports two analytic studies: **study 1** was a retrospective observational study to compare median weight trajectories of healthy, neutered, client-owned domestic shorthair (DSH) kittens in optimal body condition with available growth standards for sexually intact kittens [16]; **study 2** was an analysis of new data, from a previously-published randomised trial [9], comparing growth-related measures at four time points between pairs of sexually-intact and neutered female siblings from litters of DSH cats in a research population. The objective of **study 1** was to determine whether neutering changes patterns of healthy weight gain during growth, whilst the objective of **study 2** was to gain further insight into the causes of any neutering-associated changes in weight gain.

### Study populations

Data for **Study 1** were derived from the clinical records of client-owned DSH kittens attending Banfield^®^ Pet Hospital (BANFIELD), a network of over 900 primary care veterinary hospitals located mainly in the USA. Data were collected between April 1994 and November 2016, with 77% of data points relating to 2004 onwards. Bodyweight is routinely measured during consultations, whilst birth date and breed data are supplied by owners when the pet is first registered, but not independently verified. Data for **Study 2** were derived from a previous trial involving pairs of 11-week-old female DSH littermates housed at the Waltham Petcare Science Institute (WALTHAM) in the UK. The original study was reviewed and approved by the Waltham Ethical Review Committee and complied with UK Home Office regulations. Full details of the study population and experimental design have been reported previously [9]; briefly, 11 littermate pairs were randomly assigned to either a neutered (neutered at 19 weeks old) or a sexually-intact (kept sexually intact) groups, and were offered free access to a dry diet until the age of 1 year. The kittens were group-housed for socialisation during the day and individually housed overnight. Kittens were fed a nutritionally-complete, commercial dry diet formulated to meet the nutritional requirements for gestation, lactation and growth and confirmed by full nutritional analysis (moisture 7·5 g, protein 33·1 g, fat 20·8 g, ash 7·35 g, non-free extract 31·4 g, predicted metabolisable energy (ME) 1628·4 kJ/100 g as fed).

### Data extraction and eligibility criteria

For **Study 1**, the BANFIELD medical records database was searched for weight measurements from DSH cats under 130 weeks (2.5 years) of age, calculated from measurement date and date of birth. For any individual recorded as having been neutered, the date for the procedure was required. Given that neuter dates were only available for cats where castration or ovariohysterectomy was performed at a BANFIELD hospital, those where procedures were performed elsewhere were excluded from the dataset. Additional eligibility criteria were applied at this stage, consistent with those used when creating the growth standards, as described elsewhere [16]. Data were only included from cats attending routine preventative healthcare visits (these were determined by Banfield’s visit type classification and included reasons such as vaccination, deworming and check-ups), or visits where the diagnosis was recorded as ‘healthy pet’. Measurements were excluded from the dataset if the recorded bodyweight had been rolled over from a previous visit (which occasionally happens when weigh scales were unavailable at the time the kitten is checked in for their appointment), or if the medical records indicated that the cat had been weighed whilst still in a pet carrier. Also excluded, were all observations from cats where the recorded sex was unclear (e.g., a cat recorded as male, but who had undergone an ovariohysterectomy), and data from cats diagnosed with a health condition before 4y of age associated that might either have affected their growth or might have caused weight loss or gain after skeletal maturity. Examples included diabetes mellitus and dwarfism. Finally, only data points were included from cats that had received a body condition rating of ‘normal’ or ‘ideal’ at one or more visits between the ages of 78 weeks and 130 weeks (1.5 to 2.5 years), which was taken as an indicator of having optimal body condition in young adulthood, and had never received an abnormal body condition rating (e.g., very thin, thin, heavy, overweight, markedly obese) at any point up to the age of 208 weeks (4 years). The restriction to healthy kittens of good body condition score ensured that the study was examining patterns of healthy growth, on the same basis as the kitten growth curves were constructed. The dataset was then restricted to kittens with at least one recorded bodyweight remaining between the ages of 5 weeks and 91 weeks. All available data on the 11 cat pairs in **Study 2** was used, meaning that no special extraction or eligibility criteria were needed.

### Generation and recording of clinical data

The methods for recording signalment data (date of birth, breed and sex), bodyweight, neuter status, neuter date and clinical diagnoses in the BANFIELD clinical records used in **Study 1** have been described previously [15]; the methods for assessing and recording body condition score (BCS) at BANFIELD, and how this was treated when preparing the dataset, have also been described for dogs [18]; exactly the same approach was taken for handling BCS in cats [16]. Briefly, BCS was mapped onto a 3-category scale (‘thin’, ‘normal’, or ‘heavy’) for the purposes of the data extraction and analysis. If BCS was unavailable, but had been recorded at a previous or subsequent consultation where the bodyweight was within ±5% of the weight recorded at that visit, then that BCS replaced the unknown one [18].

For the randomised trial data used in Study 2, body composition, morphological measurements and fasting blood samples were taken at 11, 18, 30 and 52 weeks of age, with neutering taking place at 19 weeks of age. Bodyweight was measured weekly, but only the measurements from 11, 18, 30 and 52 weeks were utilised for the current study. As described previously [9], lean mass and fat mass were determined using dual-energy X-ray absorptiometry (DEXA), whereby kittens were sedated, placed in lateral recumbency and body composition was analysed using a Hologic QDR-1000 W pencil beam dual-energy X-ray absorptiometer (Hologic, Inc., Waltham, MA, USA). Zoometric parameters were measured in fasted, conscious and non-sedated cats. Measurements were taken from their left-hand side, whilst they were standing with legs perpendicular to the ground and with their head in an upright position and looking forward. Height was measured at the points of the scapula; length from manubrium of the sternum to a parallel point below the anus; girth around the narrowest point of the waist; ribcage at the deepest part of the thorax (approximately the point of the sternum and eighth rib); chest depth at mid-thorax, from spine to the deepest part; elbow width across the condyle of the humerus; forelimb length from olecranon to carpus; and hindlimb length from patella to tarsus. Length was measured with a purpose-adapted measuring stick; girth and ribcage were measured with a graduated measuring tape; whilst chest depth, forelimb and hindlimb were measured with digital callipers. Fat mass, lean mass and bodyweight were recorded in kg, whilst all zoometric measurements were measured in cm.

### Data handling and statistical analysis

#### Sample size

For **Study 1**, the aim was to include as many cats as possible that met the eligibility criteria, so a formal sample size calculation was not performed. However, the resulting dataset was of comparable size to that used to create the growth standards [16]. The data used in **Study 2** were from a historic trial, and the method used to decide sample size was not reported; however, the fact that statistical differences were found with some of the endpoints [9], suggests that the study was not universally underpowered.

#### Data cleaning

For the **Study 1** dataset, several data subsets were constructed for male and female cats, according to age of neutering. The neutering age splits used corresponded to the lower quartile, median and upper quartile of the neutering ages observed for that sex across all castration and ovariohysterectomy procedures of DSH cats between April 1994 and November 2016 (regardless of whether the cat was eligible for the study population). Each subset comprised all data from sexually-intact cats of the relevant sex up to the lower end of the group’s neuter age range, then subsequently only cats neutered after the lower end but before the upper end of the group’s neuter age range. This resulted in 8 data subsets, comprising male and female subsets for each of the 4 neutering age ranges. The only data cleaning prior to this step was the removal of weights which had been rounded to the nearest whole unit (pounds), as previously described [9,16]. Subsequently, each of the data subsets was cleaned independently, again using a similar approach to that used for the dataset of the growth standards [16]. Briefly, extreme outliers (i.e., bodyweight entries >3 times the median value for cats >9mo age) were removed, as were population outliers, identified as data points outside of loess-smoothed curves fitted through the outlier limits of bodyweight for each of 50 equally-sized age groups. The outlier limits were defined as 175% of the upper and lower whiskers of a box-and-whisker plot. Additional data cleaning measures were then implemented for cats with repeat visit data, to remove weights that were deemed to be implausible given the remaining weight trajectory for that individual.

When considering the data for **Study 2**, it was assumed that for height, chest depth, length, elbow width, forelimb and hindlimb, a large drop (>10%) over successive time points was unlikely, since it would be expected that these measurements should normally increase due to growth over the time intervals used in the trial. Pairs of data points displaying such a drop were identified and, for each pair, the data point with the largest z-score (calculated over all instances of that measurement at that time point) was removed. Weight, girth, ribcage, fat and lean were not cleaned in this way because these measures could be influenced by changes in adiposity, meaning they could not necessarily be assumed to be increasing. Instead, they were examined visually for any obviously erroneous data.

#### Statistical analysis—Study 1

For the BANFIELD client-owned cats, the effect of neutering on bodyweight (analysis 1) was firstly assessed by modelling the growth trajectories separately in each of the data subsets, and then comparing the average growth trajectories with growth standards for sexually-intact cats [16]. The same modelling of growth trajectories was used as for the growth standards [16]; briefly, the trajectories were modelled using generalised modelling for location, shape and scale (GAMLSS), specifically the BCCG (Box-Cox Cole-Green) model, which is a semi-parametric technique allowing the central tendency, spread, skewness and kurtosis of the data to be estimated as smooth functions of the predictor variable(s). The variables used were age (raised to the power of 0.1 which initial data exploration suggested improved the model fit, as it did for the growth standards [16]) and bodyweight. The median and interquartile (25% and 75%) centiles were then extracted from each model and converted to z-scores according to the growth standards. The z-score transformation was used because it makes interpretation of different growth patterns more straightforward, since the standard centiles of the growth standards become equally spaced horizonal lines, with the median centile line lying along the x-axis. Further, the transformation converts a trajectory fully following the growth standards to a horizonal line, whilst positive and negative gradients depict faster and slower growth, respectively. These z-score transformations were displayed for each data subset, alongside the known neuter age-ranges, to enable visual inspection of any disturbances to average growth after neutering.

A secondary analysis (analysis 2), compared z-scores before and after neutering within individuals. For each cat in the dataset, z-scores at two visits were identified: the last visit prior to neutering and the last visit in the data post-neutering. Kittens that did not have both such visits, or where those visits were less than 3 months apart, were excluded from the analysis. This subset was analysed using a linear model, which included the post-neutering z-score as the dependent variable and pre-neutering z-score, sex and neuter group as the predictor variables. For each combination of sex and neuter group, the estimated mean difference in z-score from pre- to post-neutering was calculated for a cat at median weight (i.e., z-score equal to zero) before neutering, along with *P-*values and 95% confidence intervals. As we were making multiple comparisons, a Tukey HSD correction [23] was used to control the familywise error rate. *P* < 0.05 was considered to denote significance.

#### Statistical analysis—Study 2

The 11 morphological measurements were simultaneously analysed using a Bayesian mixed model with a multivariate endpoint. This approach was preferred to separate univariate models because it allows for correlations amongst measures and renders separate adjustments for multiple endpoints unnecessary. The dependent variables were log-transformed to improve normality and then standardised to similar scales (which can improve numeric efficiency) using a z-transformation. The model contained identical terms for all measures. Group (neutered or sexually-intact), week and their interaction were included as fixed factors, whilst litter ID and cat ID (nested in litter ID) were included as random factors. Correlation between measures was allowed at the level of both cat and litter. A normal distribution, with a mean of 0 and standard deviation of 4, was used as the prior for every endpoint; this distribution easily covered the entire set of raw data and, therefore, was not considered particularly informative; however, it still enabled the model to converge, which was not the case with a formal non-informative prior. Missing values in the dependent variables were imputed during model fitting, which ensured that other measures for that individual and timepoint could still be used. Posterior values were sampled using Markov chain Monte Carlo (MCMC); 4 chains of 10,000 values were simulated, with the first 5,000 values used as warm-up and the remaining values thinned to 1 in 5, giving a final set of 4,000 posterior samples. To assess model fit, a density trace comparing 40 posterior samples with the actual data was created for each endpoint for each week and residual plots were reviewed. Finally, estimated means and simultaneous 95% credible intervals were calculated for each measure for each group and timepoint, and for the difference between the groups in the fold-change with respect to each measure from 11 weeks (when both groups were still sexually-intact) to each subsequent timepoint. Simultaneous credible intervals are essentially multivariate intervals which are calculated to have a given probability of containing the true value of the multivariate quantity under consideration and, therefore, control for type I error.

### Software

All analyses were performed with an open-access online statistical software environment (R, version 3.6.1) [24], using the packages gamlss [25] for the GAMLSS modelling, the multcomp package [23] for multiple comparisons and brms [26] for the Bayesian modelling. Graphics were produced using ggplot2 [27].

## Results

### Sample population, data extraction and cleaning

Before creating subsets according to age at neutering, the dataset for **Study 1** comprised 43.5k bodyweights (19.1k male; 24.4k female) across 11.4k cats (5.00k male; 6.41k female); this decreased to 36.7k bodyweights (16.1k male; 20.6k female) across 10.2k cats (4.45k male; 5.71k female) after removing weights that had been rounded to the nearest whole unit. The neutering age splits were calculated from a dataset of 188k castration and 181k ovariohysterectomy operations, comprising all such operations between April 1994 and November 2016 (regardless of whether the cat was eligible for the study population), as explained previously. For male kittens, the resulting groups were <0.39y, 0.39–0.44y, 0.44–0.54y and >0.54y; for female kittens, the resulting groups were <0.40, 0.40–0.48y, 0.48–0.56y and >0.56y. For convenience, the groups are subsequently referred to as neuter groups 1 to 4 for each sex, respectively.

Table 1 shows the number of rows of data and individual cats in each neuter group at each stage in the subsequent data cleaning process, from subset creation to final datasets. Since each neuter group included data for all eligible sexually-intact kittens up to the lower end of the neuter age range, the datasets were not of equal size; the first group was notably smaller overall than the other three groups in both sexes, due to differences in the numbers of eligible cats and the distribution of visits with age (for example, some kittens did not have any visits prior to the lower boundary of the first neuter age range and, therefore, could not be used for this group). There was less variation in the number of cats remaining in each group after neutering, however, ranging from 692 to 945 in male kittens and 717 to 1,246 in female kittens.

**Table 1:** Details of the data subsets for Study 1 at each stage of data cleaning.

Sex	Neuter Group^a^	Cleaning Stage					
		Dataset Creation	Removal of Extreme Outliers	Population Level Cleaning	Removal of Duplicate Observations	Within-Cat Cleaning	FINAL DATA
M	1 (up to 0.39y)	3,541 rows 775 cats	3,541 rows 775 cats	3,492 rows 774 cats	3,490 rows 774 cats	3,039 rows 712 cats	3,039 rows 712 cats
	2 (0.39–0.44y)	7,875 rows 2,641 cats	7,876 rows 2,641 cats	7,695 rows 2,605 cats	7,689 rows 2,605 cats	5,961 rows 2,141 cats	5,961 rows 2,141 cats
	3 (0.44–0.54y)	9,064 rows 3,038 cats	9,063 rows 3,038 cats	8,862 rows 2,995 cats	8,855 rows 2,995 cats	6,863 rows 2,453 cats	6,863 rows 2,453 cats
	4 (over 0.54y)	10,149 rows 3,888 cats	10,148 rows 3,888 cats	9,925 rows 3,825 cats	9,916 rows 3,825 cats	7,198 rows 3,007 cats	7,198 rows 3,007 cats
F	1 (up to 0.40y)	4,780 rows 1,032 cats	4,779 rows 1,032 cats	4,692 rows 1,029 cats	4,687 rows 1,029 cats	4,061 rows 952 cats	4,061 rows 952 cats
	2 (0.40–0.48y)	10,625 rows 3,319 cats	10,622 rows 3,319 cats	10,324 rows 3,267 cats	10,310 rows 3,267 cats	7,987 rows 2,662 cats	7,987 rows 2,662 cats
	3 (0.48–0.56y)	11,091 rows 3,789 cats	11,090 rows 3,789 cats	10,730 rows 3,712 cats	10,772 rows 3,712 cats	8,061 rows 3,010 cats	8,061 rows 3,010 cats
	4 (over 0.56y)	13,123 rows 4,870 cats	13,122 rows 4,870 cats	12,729 rows 4,782 cats	12,719 rows 4,782 cats	9,281 rows 3,785 cats	9,281 rows 3,785 cats

^a^ Since each neuter group contains data for sexually intact individuals up to the lower end of the neuter age range, the neuter groups are not disjoint.

The original raw data for **Study 2** had 3 missing values (fat mass, 2; lean mass, 1), for unknown reasons, whilst a further 6 observations (chest depth, 3; height 2; hindlimb, 1) were removed during data cleaning.

### Statistical analysis—Study 1

**Fig 1:**
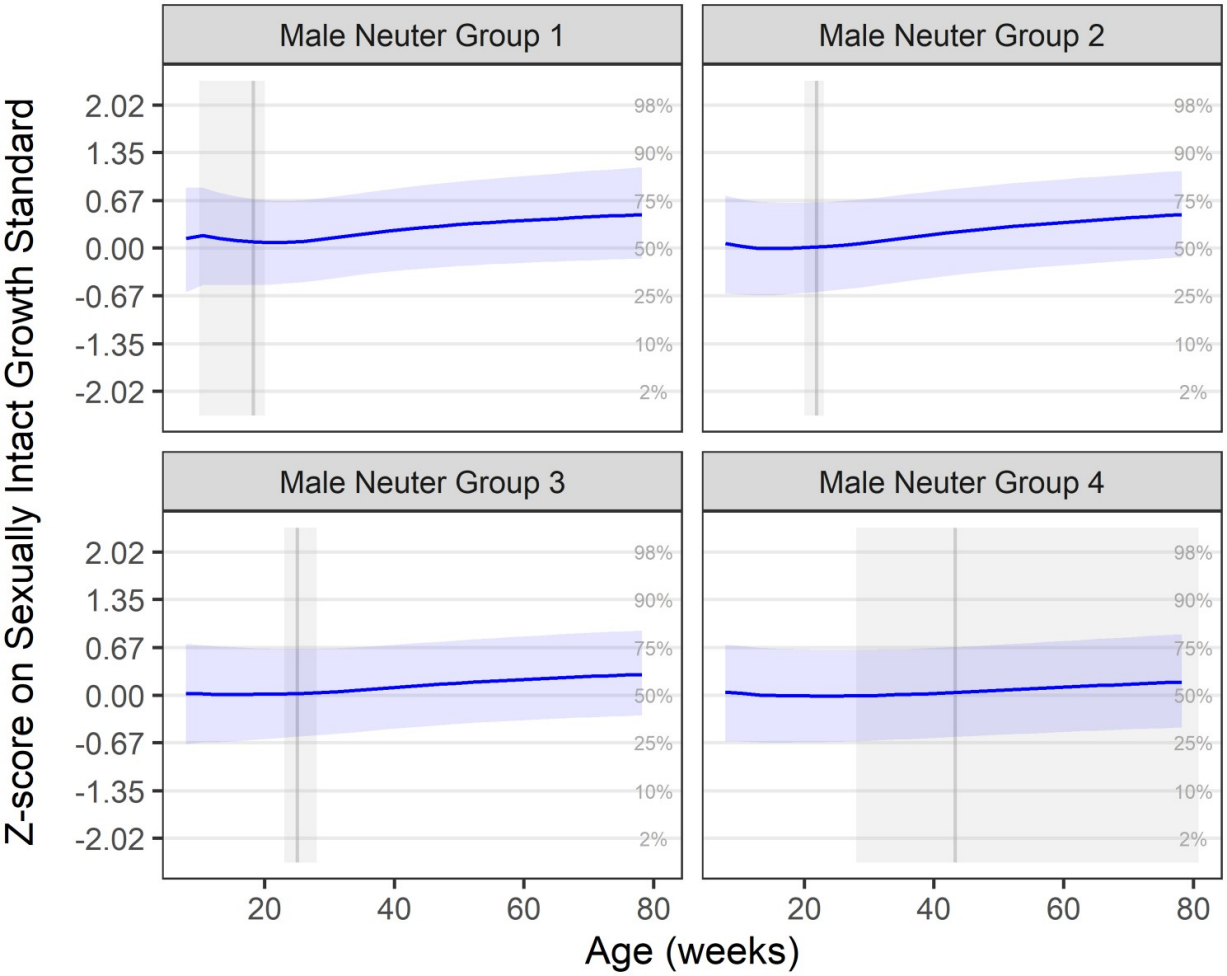
Median male growth trajectory by neuter group on z-score scale. Neuter Groups 1–4 represent, respectively, neutering ages of up to 20 weeks (0.7k cats), 20–23 weeks (2.1k cats), 23–28 weeks (2.5k cats) and >28 weeks (3.0k cats). Groups calculated from the lower quartile, median and upper quartile of ages at all neutering procedures performed on DSH cats between April 1994 and November 2016. The solid blue line represents the mean trajectory, whilst the blue-shaded ribbon represents the interquartile range. The grey shaded area represents the neutering age range for the group, and the solid grey vertical line shows the median observed neutering age. Grid lines represent the standard growth centiles, such that a growth trajectory following the centile curves would be horizonal in these plots. In all groups, there was an upwards inclination in growth trajectory, which was most marked in neuter groups 1 and 2.

**Fig 2:**
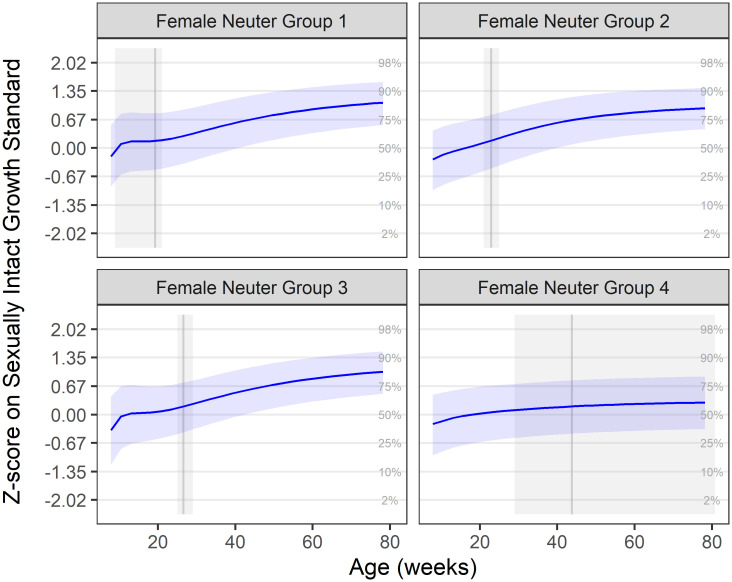
Median female growth trajectory by neuter group on z-score scale. Neuter Groups 1–4 represent, respectively, neutering ages of up to 21 weeks (1.0k cats), 21–25 weeks (2.7k cats), 25–29 weeks (3.0k cats) and >29 weeks (3.8k cats). Groups were calculated from the lower quartile, median and upper quartile of ages at all ovariohysterectomy procedures performed on DSH cats between April 1994 and November 2016. The solid blue line represents the mean trajectory, whilst the blue-shaded ribbon represents the interquartile range. The grey shaded area represents the neutering age range for the group, and the solid grey vertical line shows the median observed neutering age. Grid lines represent the standard growth centiles, such that a growth trajectory following the centile curves would be horizonal in these plots. In all groups, there was an upwards inclination in growth trajectory, which was marked in neuter groups 1–3, but only modest in neuter group 4.

The aim of this part of the analysis was to compare the patterns of growth (as measured by bodyweight) in kittens, neutered at four different age periods, with previously calculated growth standards based on sexually intact kittens. In analysis 1, the median growth trajectories for each neuter group were calculated and expressed as z-scores of the growth standards. These are shown in Figs 1 and 2, for male and female kittens, respectively; these same median growth trajectories are also depicted relative to the centile standards in the (S1 and S2 Figs). In all neuter groups of both sexes, median growth trajectory was close to the zero z-score line (representing the median growth standard) before neutering, but there was an upwards inclination in trajectory afterwards: for male kittens, the upwards inclination in median growth was 0.34, 0.41, 0.27 and 0.14, in neuter groups 1, 2, 3 and 4, respectively, by the end of the growth period; for female kittens, the upwards inclination in median growth was 1.27, 1.21, 1.39 and 0.51, in neuter groups 1, 2, 3 and 4, respectively, by the end of the growth period. For all ages, the upwards inclination was more marked in female compared with male kittens. However, in both sexes, the inclination was less steep in kittens neutered later.

**Fig 3:**
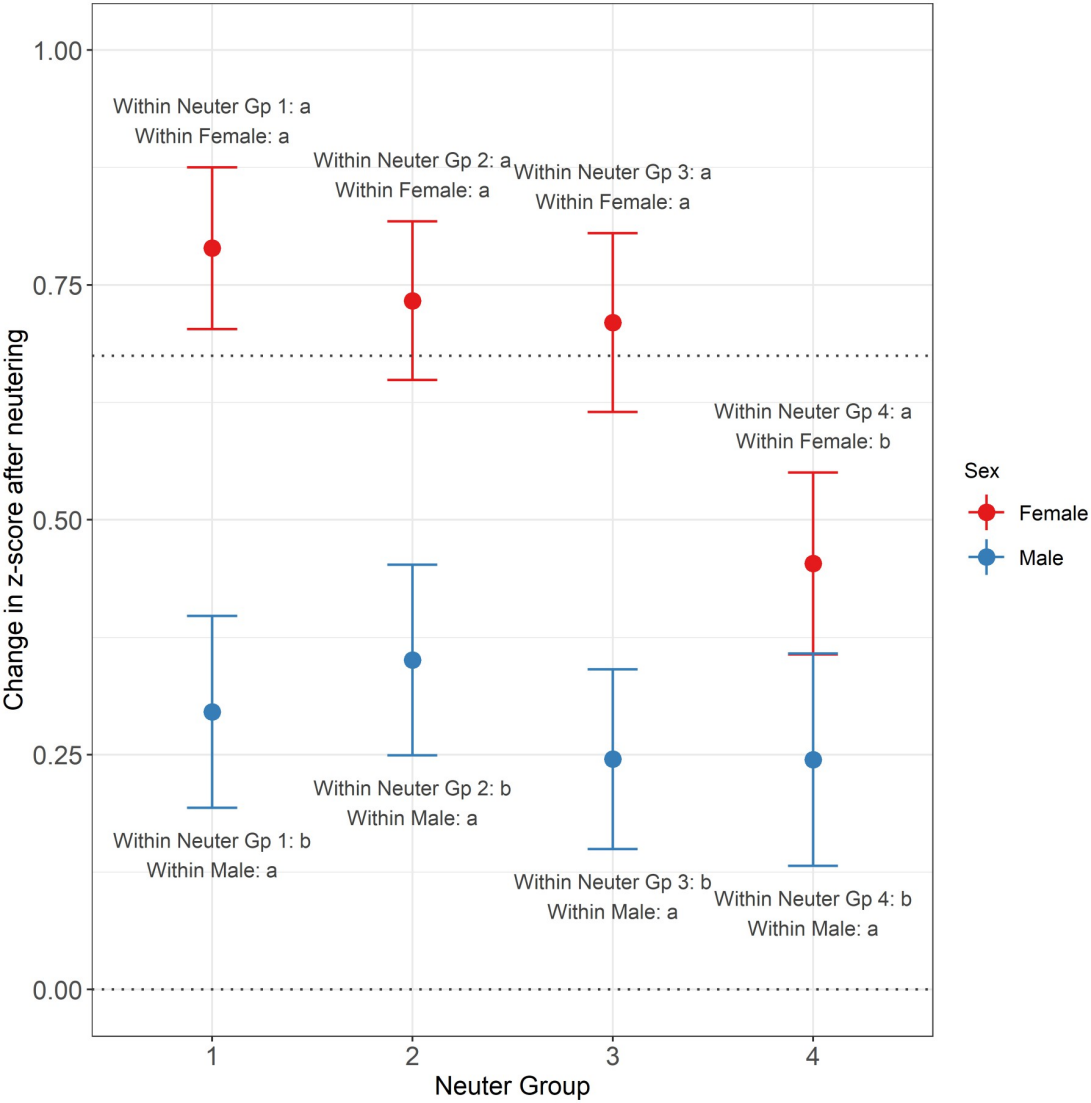
Estimated change in z-score post-neutering for a cat at median weight. Shown for different combinations of sex (female: Red; male: Blue) and neuter group, with 95% confidence intervals and Tukey post-hoc comparison groups. Neuter Groups 1–4 represent, respectively, neutering ages representing quartiles in the general neutered population–up to 20 weeks (448 cats), 20–23 weeks (451 cats), 23–28 weeks (507 cats) and >28 weeks (364 cats) for males, and up to 21 weeks (628 cats), 21–25 weeks (653 cats), 25–29 weeks (515 cats) and >29 weeks (497 cats) for females. The lower horizontal dotted line shows the point of zero change (meaning the cat remained on its initial centile line) and the upper horizontal dotted line represents an increase equal to one standard growth centile interval. The annotations give the Tukey post-hoc comparison groups, which indicate whether differences exist between estimated means for different combinations of neuter group and sex. Only comparisons between neuter groups of the same sex, and between sexes within the same neuter group were tested, and the groups within the relevant neuter group and relevant sex are as indicated in the annotations; within sets of means sharing the same neuter group or sex, those with different letters/numbers for that sex/group are significantly different at the 5% significance level. In all groups, there was a significant increase in z-score post neutering, and this was significantly larger in female than male.

The purpose of analysis 2 was to compare the difference in z-score before and after neutering within individuals. Fig 3 shows the estimated change in z-score post-neutering for a cat at median weight, for different combinations of sex and neuter group, together with 95% confidence intervals and Tukey post-hoc comparison groups. The number of cats in each of these combinations varied from 364 (Male, Neuter Group 4) to 653 (Female, Neuter Group 2).

On average, there was a significant increase in z-score after neutering for all neuter groups and sexes (all *P*-values < 0.001), with this increase being larger for females than for males. It was also apparent that the latest neutered groups tended to show a directionally smaller increase than the earlier neutered groups, as noted in the population level analysis, although this was only significant for female neuter group 4 compared to earlier neutered groups.

### Statistical analysis—Study 2

The purpose of this second study was to compare growth-related measurements between pairs of sexually-intact and neutered female DSH littermates, in order to gain insights into the nature of growth changes associated with neutering; this was achieved with a Bayesian mixed model fitted to 11 morphological measurements taken at 4 time points. Overall, the fit of this model was deemed to be satisfactory, with the results of the posterior predictive checking of the model shown in S3 Fig. Posterior density traces coincided well with the observed traces for most of the measure-timepoint combinations; the main exceptions were chest depth and elbow width, at 10wk, and length at 18wk, where there were relatively small divergences.

**Fig 4:**
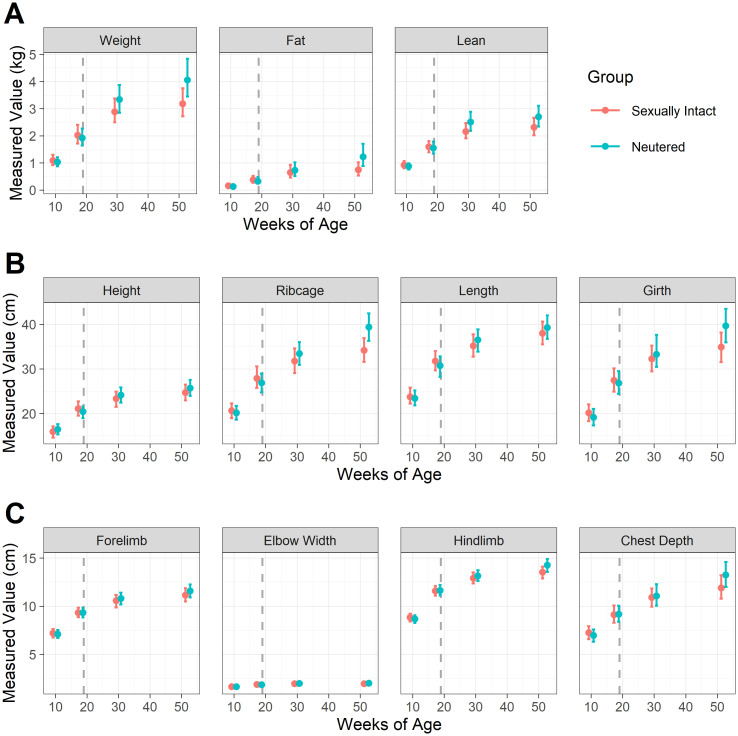
Estimated means and simultaneous 95% credible intervals for each measure, for each group and timepoint, shown on the original scales. The 11 sexually-intact cats are shown in red, whilst the 11 paired cats that were neutered are shown in blue; the grey dashed line indicates age of neutering. Panel A shows mass measurements, whilst and panels B and C show zoometric measurements. Weight, fat mass and lean mass measured by dual-energy X-ray absorptiometry (Hologic QDR-1000 W; Hologic, Inc., Waltham, MA, USA); height measured at the points of the scapula; ribcage measured at the deepest part of the thorax; length measured from manubrium of the sternum to a parallel point below the anus; girth measured around the narrowest point of the waist; forelimb bone measured from olecranon to carpus; elbow width, measured across the humeral condyles; hindlimb bone measured from patella to tarsus; chest depth measured mid-thorax from spine to the deepest part.

**Fig 5:**
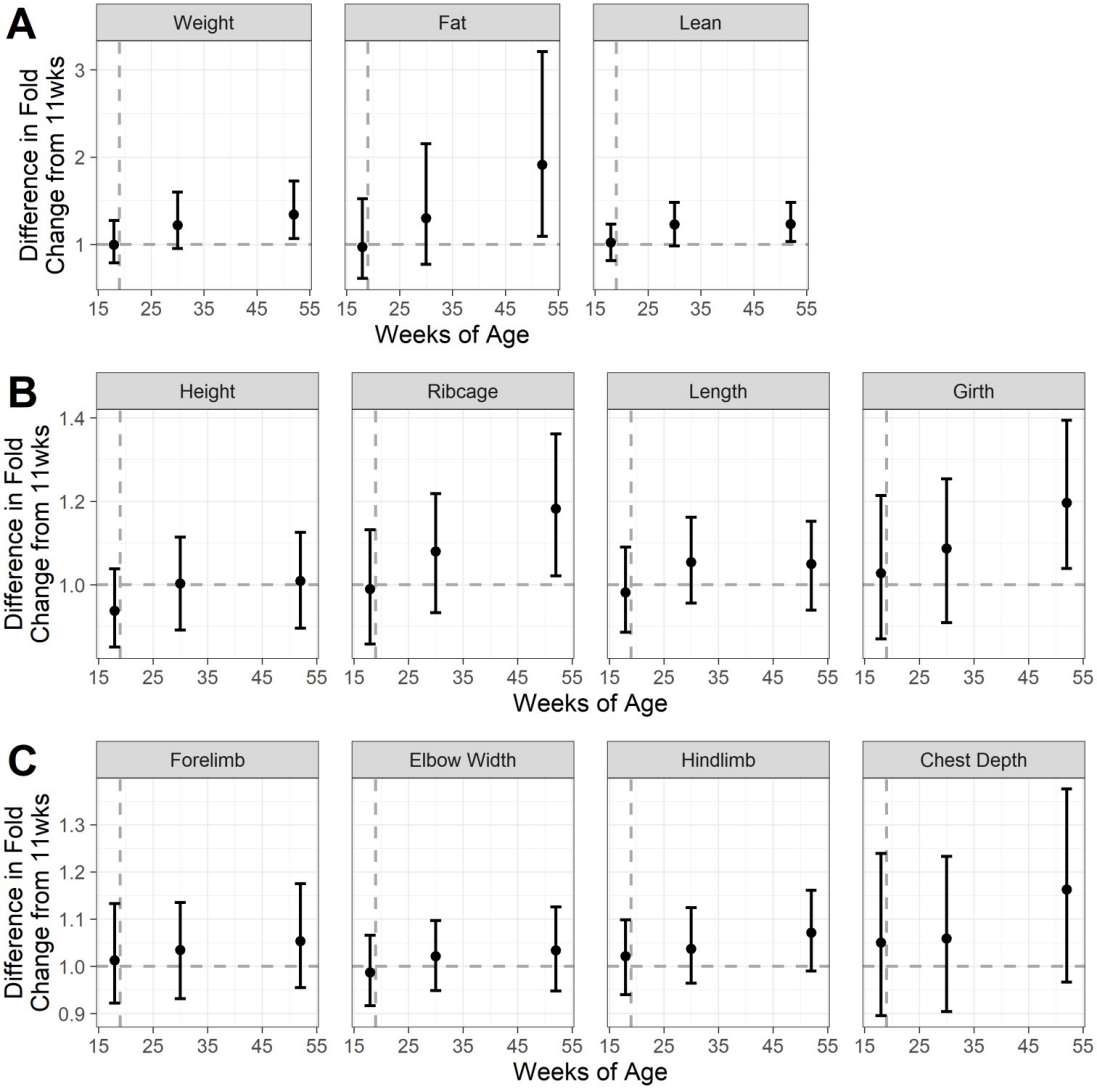
The estimated mean and 95% simultaneous credible intervals (difference between sexually-intact and neutered kittens (11 cats per group), in the fold-change for each measure, between 11 wk (when both groups were still sexually intact) and each subsequent timepoint. Black dots represent the mean values, whilst error bars represent the 95% simultaneous credible intervals; the vertical grey dashed line indicates age of neutering. Differences are shown as the fold change of the neutered group divided by the fold change of the sexually-intact group, so that values >1 (shown by the horizontal dashed line) indicate that the neutered group had a greater difference in fold change. Panel A shows mass measurements, whilst and panels B and C show zoometric measurements. For details of the measurements see the legend for Fig 4.

**Fig 6:**
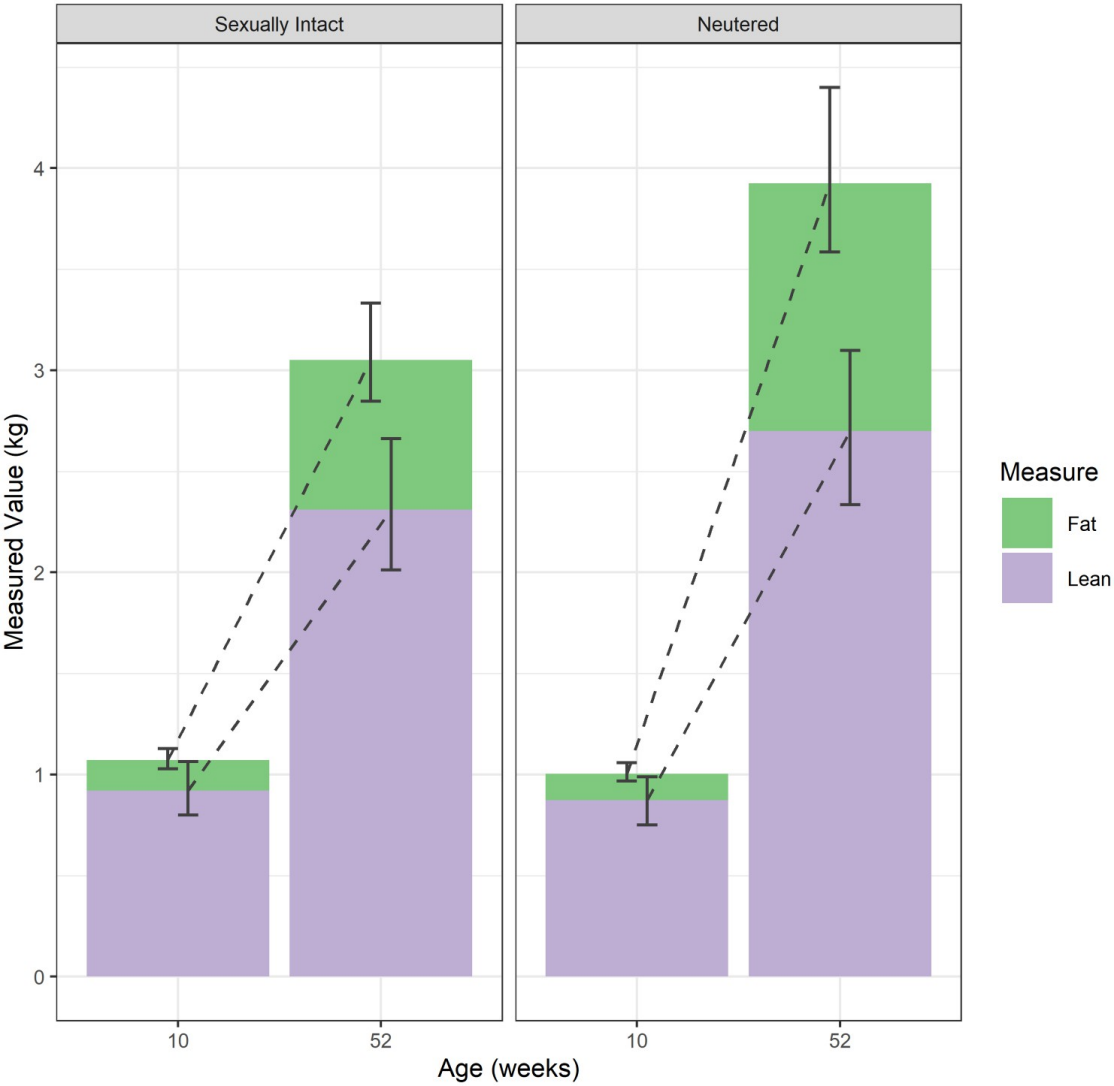
Estimated mean fat mass (green) and lean mass (lilac) at the initial and final measurement points for the sexually-intact and neutered kittens (11 cats per group). The intervals shown are simultaneous 95% credible intervals, whilst the green and lilac portions of each column represent fat and lean mass, respectively.

Fig 4 shows estimated means and simultaneous 95% credible intervals for each measure, in each group (sexually-intact and neutered) and at each timepoint; Fig 5 shows the difference between the groups in the fold-change from 11wk (when both groups were still sexually intact) to each subsequent timepoint; and Fig 6 shows the proportional change in body composition between week 10 and week 52. Full details of means and 95% credible intervals for the values at each timepoint, the fold change from 10wk-old and the difference in the fold change between groups for all endpoints are given in (S1 Table). By 52wk, neutered kittens were heavier (mean difference 1.34, 95-CI: 1.07–1.72), had more fat mass (mean difference 1.91, 95-CI 1.09–3.21) and more lean mass (mean difference 1.23, 95-CI: 1.03–1.48) compared with sexually-intact kittens. Abdominal girth (mean difference 1.20, 95-CI: 1.04–1.39) and rib cage length (mean difference 1.18, 95-CI: 1.02–1.36) were also greater, but there were no differences in other zoometric measurements.

## Discussion

In the current study, we have compared patterns of growth in neutered healthy domestic shorthair kittens with growth depicted by standards created using data from sexually-intact kittens. Our ultimate aim was to inform guidelines to support the usage of the standards in neutered kittens. Neutering was associated with an upwards deviation in growth, which ranged from 0.14 of a centile unit in late-neutered male kittens to 1.4 centile units in early-neutered female kittens. Thus, the physiological changes resulting from neutering provoke more rapid weight gain such that cats are likely to be heavier when they reach skeletal maturity. It is noteworthy that these changes are more marked than was seen in a recent study in dogs [18]; although early neutering in dogs led to a modest increase in growth rate, and later neutering a modest decrease in growth rate, these changes were slight and within a single centile unit. The reasons for this difference between species are not known and require further study.

As the purpose of the study was to examine weight changes post-neutering for cats showing apparently healthy growth, cats eligible for inclusion in the first part of the study had never received an abnormal body condition rating (including heavy, overweight or markedly obese) before the age of 2.5y. Therefore, the impact of neutering, and especially early neutering, on bodyweight calculated here is likely to be an under-estimate for the population as a whole. The results of the second part of this study suggest that the weight gain after neutering mainly (but not exclusively) comprised adipose tissue, which is consistent with previously-reported associations between neutering and obesity [7,8]. On the face of it, the results from these two studies contradict one another given that cats were reportedly in ideal condition, based on body condition assessments. However, this difference is likely to be due to the inaccuracy of body condition assessments rather than the fact that there was no gain in adipose tissue. Prior to 2010, veterinarians scored body condition subjectively using a 3-category system (underweight, normal, overweight) whilst, after this time, body condition was scored using a 5-point BCS [18]. Although BCS correlates moderately well with body fat mass measured by DEXA, it is only semi-quantitative, insensitive and only been validated in adult cats [17,28]. Further, scores are known to be affected by operator expertise, whereby BCS from veterinarians correlate better than for veterinary technicians and observers without training [29]. In previous studies, one unit on the 9-point system equates to a difference of approximately 10% (but up to 15%) bodyweight [17,28], whilst half a unit on the 5-unit system equates with an approximately 7% change in body fat percentage [29]; it is likely that the both the 3-category system and 5-point BCS used in the current study are even less sensitive (given fewer scores with which to differentiate cats). Therefore, slight increases in adipose tissue might well have been missed with both systems. Increases in adiposity might also have been obscured by other concurrent changes in body composition, given that a smaller, but nonetheless significant, increase in lean mass was also seen after neutering in the second study whilst some changes in body shape were also observed (e.g., increased abdominal girth and rib cage length). Alternatively, the bulk of the increase in adipose tissue could have been in an intra-abdominal, rather than subcutaneous, position. It is possible that both such changes (accumulation of abdominal fat, concurrent increase in lean mass) made an obvious change in BCS was less evident.

The changes in lean mass observed after neutering in the current study contrast with those of other studies, where no change in lean mass was seen [30]. Increased lean mass was also not reported in the original paper about this study; only percentage lean was included [9]. This difference may be explained by the fact that percentage fat mass is negatively correlated with percentage lean mass such that as body fat percentage increases, lean tissue percentage decreases. Given that the difference in the fat mass increase between 10 and 52 weeks of age was of a much greater magnitude in the neutered kittens than the difference in the lean mass increase (1.91 vs. 1.23 times greater in the neutered kittens), the increase in lean mass was obscured. This effect would only be exacerbated if studies were insufficiently powered. The nature of this lean mass increase is unclear, not least because the lean mass measurement detected by DEXA not only includes muscle but other soft tissues such as abdominal organs [31]. Therefore, it is possible that the increase in lean mass might have included increased abdominal organ size; it has been found previously that the growth rate of pigs is positively correlated with abdominal organ mass [32]. Other body composition techniques, such as computed tomography, would be required to differentiate changes in muscle mass from organ mass.

The finding of increased fat and lean mass is consistent with the effects of overnutrition in other species. For example, children with obesity are often tall for age, and there is an increase in lean mass as well as fat mass [33]. Further, an increased plane of nutrition in production animals also results in increases in stature, body fat and lean mass [33]. Such overnutrition is likely to be the direct result of physiological changes that occur after neutering, most notably increases in food intake which, as previously reported [8,9], result from the direct result of decreased sex hormone production, not least given that the effect can be reversed by administration of oestradiol [30]. A further consideration is the complex interaction between sex, hormones, growth and adiposity; as mentioned above, children with obesity are often taller than their peers in pre-puberty but have an earlier onset of puberty with less of a ‘growth spurt’ at this stage [34]. This is the result of early growth plate maturation, which is triggered by the hormonal changes that occur at puberty. Therefore, it is plausible that prepubertal neutering in kittens might delay epiphyseal growth plate closure, given an absence of the usual hormonal changes at this time, meaning an increase in long bone length and a taller stature compared with sexually-intact cats or those neutered after puberty. However, the results of the current study are not consistent with such a hypothesis because there were no significant differences in zoometric measurements pertaining to length (elbow width, forelimb, height and length).

The findings of the current study, whereby neutering in cats was associated with weight gain and increased adipose tissue mass, is similar to the findings of other studies [7–9]. Previous studies have suggested that these changes are the result of increased voluntary food intake [7–9], possibly, with a concurrent decrease in physical activity and energy expenditure [8]; the latter is supported by findings from a recent study, examining growth patterns in male and female kittens, whereby neutering was associated with decreased energy requirements for growth in both male and female kittens [35]. Neutering had a greater impact on the growth trajectory of female compared with male kittens, and this has also been reported previously [6]. Although the reason for the difference between the sexes is not known, it might be due to effects of the absence of female sex hormones since oestradiol administration both to male and female cats abrogates the increased food intake that occurs in both male and female cats after neutering [30]. Experimental studies in rodents have suggested that this effect might be mediated through modulation of cholecystokinin (CCK)-dependent satiety signals [36,37]. However, further studies would be required to confirm whether the same effect occurs in cats.

In recent, years the pre-pubertal neutering of cats has been promoted within the veterinary profession [19] mainly because of perceived benefits such as population control, as well as reducing development of unwanted male characteristics and behaviour (unwanted urination aggression, roaming and marking) and a decreased risk of some types of neoplasia [19,20,22]. Perceived barriers to early neutering, such as surgical and anaesthetic risk, have also been addressed with improvements in anaesthetic and surgical techniques, and recovery has been shown to be quicker [3]. Further, concerns over increased risk of developmental (orthopaedic or urogenital) disorders have not proved to be a major concern, at least in cats [2]. Thus, on a risk-benefit analysis, pre-pubertal neutering has largely been promoted as positive both ethically and on a welfare basis. However, in these discussions, relatively little consideration has been given to the possible effect of early neutering on increased adiposity and obesity. The results of the current study, which demonstrate that early neutering leads to a more profound increase in weight than neutering later on, especially in female kittens, should prompt a reconsideration of the risk-benefit analysis. Although it could be argued that any possible increased risk is easily addressed by recommending that owners limit food intake after neutering, this might be more difficult to implement in clinical practice. Obesity is a complex, multifactorial disease, associated with comorbidities, shortened lifespan and poorer quality of life [38]. Despite increasing awareness, prevalence has still increased dramatically recently, and strategies to manage it are imperfect [38]. Therefore, if early neutering is to be promoted by veterinarians, much better guidance on management of weight post-operatively is essential. The authors recommend regular and frequent weight checks after neutering, ideally at least every month, until the impact on weight status is established. The recently-developed growth charts [16] could be used to identify changes in growth trajectory, and adjustments to food intake can then be made, for example if centiles are crossed in an upwards direction. Such an approach would increase the likelihood of a cat reaching skeletal maturity still at a healthy weight, but it might not be sufficient to prevent obesity later in life, not least because weight is gained progressively throughout early-to-mid adult life [39]. A lifelong programme of weight monitoring would be needed to address this, as outlined previously [40]. Finally, close attention should be placed on nutritional management post-neutering, as recently reviewed [41]; recommendations would include avoiding *ad libitum* feeding, accurately calculating energy requirements (to 314 kJ [75 kcal] per kg^0.67^ per day) and feeding diet formulated for neutered cats [41]. Given the use of electronic patient records in study 1, it was not possible to determine whether any such strategies had been adopted in any of the cats that participated, and any impact it might have had. It is also not possible to determine whether such nutritional strategies post-neutering would have an effect on adipose tissue deposition given that, in study 2, all cats were offered free access to dry food during their growth phase.

The study has some limitations that should be acknowledged, many of which have already been reported previously [16,18]. First, the population used for study 1 was restricted to cats in optimal body condition, so as to fulfil the first aim of the study of informing recommendations around using the growth standards in neutered kittens. Given that the magnitude of weight gain in kittens developing obesity during growth would be even more than seen in the kittens we studied, it is likely that we have under-estimated the effect of neutering on body fat mass in the population as a whole. Second, data from pet cats were collected retrospectively, over an extended period, from a network of veterinary hospitals across North America. This might have introduced variability, because of changes to practice protocols and data collection, as discussed elsewhere [16,18], and the results from these cats might not be directly comparable with the British research colony cats used in the second study. Finally, only female kittens neutered at 19 weeks were assessed in study 2 meaning that we were unable to assess the effects in male kittens and also differences in neutering at different ages. Further studies would be required both to confirm and extend the current findings.

## Conclusion

Neutering in pet kittens leads to an upwards inclination in growth, with the effect being greater in female than male kittens and more profound in kittens neutered earlier in life. Changes in weight after neutering are mainly the result of increased adipose tissue, although there is a lesser gain in lean mass. Veterinarians should be aware of the potential impact that neutering, especially when undertaken early in life, has on gain of adipose tissue. As well as considering this in any risk-benefit analysis about age of neutering, close-monitoring of bodyweight should be undertaken post-surgery, to identify and rectify unwanted weight gain.

## Supporting information

S1 Fig Median male growth trajectory by neuter group on kg scale. Neuter Groups 1–4 represent, respectively, neutering ages of up to 20 weeks (0.7k cats), 20–23 weeks (2.1k cats), 23–28 weeks (2.5k cats) and >28 weeks (3.0k cats). Groups calculated from the lower quartile, median and upper quartile of ages at all neutering procedures performed on DSH cats between April 1994 and November 2016. The solid blue line represents the mean trajectory, whilst the blue-shaded ribbon represents the interquartile range. The grey shaded area represents the neutering age range for the group, the solid grey vertical line shows the median observed neutering age and the dashed lines represent the standard growth centiles. In all groups, there was an upwards inclination in growth trajectory relative to the standards, which was most marked in neuter groups 1 and 2.
(TIF)

S2 Fig Median female growth trajectory by neuter group on kg scale. Neuter Groups 1–4 represent, respectively, neutering ages of up to 21 weeks (1.0k cats), 21–25 weeks (2.7k cats), 25–29 weeks (3.0k cats) and >29 weeks (3.8k cats). Groups calculated from the lower quartile, median and upper quartile of ages at all neutering procedures performed on DSH cats between April 1994 and November 2016. The solid blue line represents the mean trajectory, whilst the blue-shaded ribbon represents the interquartile range. The grey shaded area represents the neutering age range for the group, the solid grey vertical line shows the median observed neutering age and the dashed lines represent the standard growth centiles. In all groups, there was an upwards inclination in growth trajectory relative to the standards, which was most marked in neuter groups 1 and 2.
(TIF)

S3 Fig Forty posterior (blue) density traces compared with the observed (red) density trace for each endpoint at each timepoint for the model in Study 2. Variables are shown on the scale used for modelling, i.e., log-transformed and standardised with the z-transformation. For most measurements, posterior densities corresponded well with the respective observed densities. The main exceptions were chest depth and elbow width, at 10wk, and length at 18wk. Height at 30wk and forelimb at 52 weeks also showed some deviation, albeit smaller.
(TIF)

S1 Table Estimated means and 95% credible intervals for the values at each timepoint, the fold change from 10wk-old and the difference in the fold change between groups for all endpoints. (PDF)

## References

[1] Sánchez-Vizcaíno F, Noble PM, Jones PH, Menacere T, Buchan I, Reynolds S, et al. Demographics of dogs, cats, and rabbits attending veterinary practices in Great Britain as recorded in their electronic health records. BMC Vet Res. 2017;13:218. doi: 10.1186/s12917-017-1138-9 28693574 PMC5504643

[2] Reichler IM. Gonadectomy in cats and dogs: a review of risks and benefits. Reprod Domest Anim. 2009;44 Suppl 2:29–35. doi: 10.1111/j.1439-0531.2009.01437.x 19754532

[3] Root Kustritz MV. Pros, cons, and techniques of pediatric neutering. Vet Clin North Am Small Anim Pract. 2014 Mar;44(2):221–233. doi: 10.1016/j.cvsm.2013.10.002 24580988

[4] Panciera DL, Thomas CB, Eicker SW, Atkins CE. Epizootiologic patterns of diabetes mellitus in cats: 333 cases (1980–1986). J Am Vet Med Assoc. 1990;197(11):1504–1508. 2272886

[5] Prahl A, Guptill L, Glickman NW, Tetrick M, Glickman LT. Time trends and risk factors for diabetes mellitus in cats presented to veterinary teaching hospitals. J Feline Med Surg. 2007;9(5):351–358. doi: 10.1016/j.jfms.2007.02.004 17449313 PMC10832956

[6] Fettman MJ, Stanton CA, Banks LL, Hamar DW, Johnson DE, Hegstad RL, et al. Effects of neutering on bodyweight, metabolic rate and glucose tolerance of domestic cats. Res Vet Sci. 1997;62:131–136. doi: 10.1016/s0034-5288(97)90134-x 9243711

[7] Nguyen PG, Dumon HJ, Siliart BS, Martin LJ, Sergheraert R, Biourge VC. Effects of dietary fat and energy on body weight and composition after gonadectomy in cats. Am J Vet Res. 2004;65:1708–1713. doi: 10.2460/ajvr.2004.65.1708 15631038

[8] Vester BM, Sutter SM, Keel TL, Graves TK, Swanson KS. Ovariohysterectomy alters body composition and adipose and skeletal muscle gene expression in cats fed a high-protein or moderate-protein diet. Animal. 2009;3:1287–1298. doi: 10.1017/S1751731109004868 22444905

[9] Alexander LG, Salt C, Thomas G, Butterwick R. Effects of neutering on food intake, body weight and body composition in growing female kittens. Br J Nutr. 2011;106 Suppl 1:S19–S23. doi: 10.1017/S0007114511001851 22005425

[10] German AJ, Ryan VH, German AC, Wood IS, Trayhurn P. Obesity, its associated disorders and the role of inflammatory adipokines in companion animals. Vet J. 2010;185(1):4–9. doi: 10.1016/j.tvjl.2010.04.004 20472476

[11] Murray JK, Roberts MA, Whitmarsh A, Gruffydd-Jones TJ. Survey of the characteristics of cats owned by households in the UK and factors affecting their neutered status. Vet Rec. 2009;164:137–141. doi: 10.1136/vr.164.5.137 19188344

[12] Chu K, Anderson WM, Rieser MY. Population characteristics and neuter status of cats living in households in the United States. J Am Vet Med Assoc. 2009;234:1023–1030. doi: 10.2460/javma.234.8.1023 19366332

[13] Clark L, Seawright AA, Gartner RJW. Longbone abnormalities in kittens following vitamin a administration. J Comp Pathol. 1970;80:113–121. doi: 10.1016/0021-9975(70)90038-1 5417926

[14] Salt C, Morris PJ, Butterwick RF, Lund EM, Cole TJ, German AJ. Comparison of growth patterns in healthy dogs and dogs in abnormal body condition using growth standards. PLoS One. 2020;15:e0238521. doi: 10.1371/journal.pone.0238521 32966286 PMC7510995

[15] Serisier S, Feugier A, Venet C, Biourge V, German AJ. Faster growth rate in ad libitum-fed cats: a risk factor predicting the likelihood of becoming overweight during adulthood. J Nutr Sci. 2013;2:e11. doi: 10.1017/jns.2013.10 25191559 PMC4153074

[16] Salt C, German AJ, Henzel KS, Butterwick RF (2022) Growth standard charts for monitoring bodyweight in intact domestic shorthair kittens from the USA. PLoS ONE 2022; 17(11): e0277531. doi: 10.1371/journal.pone.0277531 36409712 PMC9678321

[17] Laflamme D. Development and validation of a body condition score system for cats: a clinical tool. Feline pract. 1997; 25: 13–18.

[18] Salt C, Morris PJ, German AJ, Wilson D, Lund EM, Cole TJ, et al. Growth standard charts for monitoring bodyweight in dogs of different sizes. PLoS One. 2017;12(9):e0182064. doi: 10.1371/journal.pone.0182064 28873413 PMC5584974

[19] Mazeau L, Wylie C, Boland L, Beatty JA. A shift towards early-age desexing of cats under veterinary care in Australia. Sci Rep. 2021;11(1):811. doi: 10.1038/s41598-020-79513-6 33462250 PMC7813850

[20] Spain CV, Scarlett JM, Houpt KA. Long-term risks and benefits of early-age gonadectomy in dogs. J Am Vet Med Assoc. 2004;224:380–387. doi: 10.2460/javma.2004.224.380 14765797

[21] Root MV. Early spay–neuter in the cat: effect on development of obesity and metabolic rate. Vet Clin Nutr. 1995;2:132–134.

[22] Stubbs WP, Bloomberg MS, Scruggs SL, Shille VM, Lane TJ. Effects of prepubertal gonadectomy on physical and behavioral development in cats. J Am Vet Med Assoc. 1996;209:1864–1871. 8944799

[23] Hothorn T, Bretz F, Westfall P. Simultaneous inference in general parametric models. Biom J. 2008; 50: 346–363. doi: 10.1002/bimj.200810425 18481363

[24] R Core Team. R: A language and environment for statistical computing. [cited 2021 February 2]. R Foundation for Statistical Computing, Vienna, Austria. http://www.r-project.org/.

[25] Rigby RA, Stasinopoulos DM. Generalized additive models for location, scale and shape. J R Stat Soc: Ser C Appl. Stat. 2005; 54: 507–554. doi: 10.1111/j.1467-9876.2005.00510.x

[26] Bürkner PC. brms: An R package for Bayesian multilevel models using Stan. J Stat Soft 2017;80:1–28. doi: 10.18637/jss.v080.i01

[27] Wickham H. ggplot2: Elegant Graphics for Data Analysis. 2nd ed. Houston, Tx, USA: Springer;. 2016.

[28] German AJ, Holden SL, Moxham GL, Holmes KL, Hackett RM, Rawlings JM. A simple, reliable tool for owners to assess the body condition of their dog or cat. J Nutr. 2006;136(7 Suppl):2031S–2033S. doi: 10.1093/jn/136.7.2031S 16772488

[29] Shoveller AK, DiGennaro J, Lanman C, Spangler D. Trained vs untrained evaluator assessment of body condition score as a predictor of percent body fat in adult cats. J Feline Med Surg. 2014 Dec;16(12):957–65. doi: 10.1177/1098612X14527472 24626465 PMC11104095

[30] Cave NJ, Backus RC, Marks SL, Klasing KC. Oestradiol, but not genistein, inhibits the rise in food intake following gonadectomy in cats, but genistein is associated with an increase in lean body mass. J Anim Physiol Anim Nutr (Berl). 2007;91(9:400–410. doi: 10.1111/j.1439-0396.2006.00667.x 17845247

[31] Buckinx F, Landi F, Cesari M, Fielding RA, Visser M, Engelke K, et al. Pitfalls in the measurement of muscle mass: a need for a reference standard. J Cachexia Sarcopenia Muscle. 2018;9:269–278. doi: 10.1002/jcsm.12268 29349935 PMC5879987

[32] Koong LJ, Nienaber JA, Pekas JC, Yen JT. Effects of plane of nutrition on organ size and fasting heat production in pigs. J Nutr. 1982;112:1638–42. doi: 10.1093/jn/112.8.1638 7097370

[33] Forbes GB. Lean body mass and fat in obese children. Pediatrics. 1964;34:308–314. 14211097

[34] Chung S. Growth and Puberty in Obese Children and Implications of Body Composition. J Obes Metab Syndr. 2017;26:243–250. doi: 10.7570/jomes.2017.26.4.243 31089526 PMC6489471

[35] Merenda MEZ, Sato J, Scheibel S, Uemoto AT, Rossoni DF, Dos Santos MP, et al. Growth Curve and Energy Intake in Male and Female Cats. Top Companion Anim Med. 2021 Aug;44:100518. doi: 10.1016/j.tcam.2021.100518 33549804

[36] Geary N, Trace D, McEwen B, Smith GP. Cyclic estradiol replacement increases the satiety effect of CCK-8 in ovariectomized rats. Physiol Behav. 1994 Aug;56(2):281–9. doi: 10.1016/0031-9384(94)90196-1 7938239

[37] Geary N, Trace D, McEwen B, Smith GP. Cyclic estradiol replacement increases the satiety effect of CCK-8 in ovariectomized rats. Physiol Behav. 1994;56:281–9. doi: 10.1016/0031-9384(94)90196-1 7938239

[38] German AJ. Weight management in obese pets: the tailoring concept and how it can improve results. Acta Vet Scand. 2016;58(Suppl 1):57. doi: 10.1186/s13028-016-0238-z 27766974 PMC5073926

[39] Serisier S, Feugier A, Venet C, Biourge V, German AJ. Faster growth rate in ad libitum-fed cats: a risk factor predicting the likelihood of becoming overweight during adulthood. J Nutr Sci. 2013;2:e11. doi: 10.1017/jns.2013.10 25191559 PMC4153074

[40] German AJ. Obesity Prevention and Weight Maintenance After Loss. Vet Clin North Am Small Anim Pract. 2016;46:913–29. doi: 10.1016/j.cvsm.2016.04.011 27255281

[41] Vendramini THA, Amaral AR, Pedrinelli V, Zafalon RVA, Rodrigues RBA, et al. Neutering in dogs and cats: current scientific evidence and importance of adequate nutritional management. Nutr Res Rev. 2020 Jun;33(1):134–144. doi: 10.1017/S0954422419000271 31931899

