# Supplementary figures and images for: Comparison of growth in neutered Domestic Shorthair kittens with growth in sexually-intact cats
Source: PLoS One. 2023 Mar 15;18(3):e0283016. doi: 10.1371/journal.pone.0283016 (PMC10016642; doi:10.1371/journal.pone.0283016)

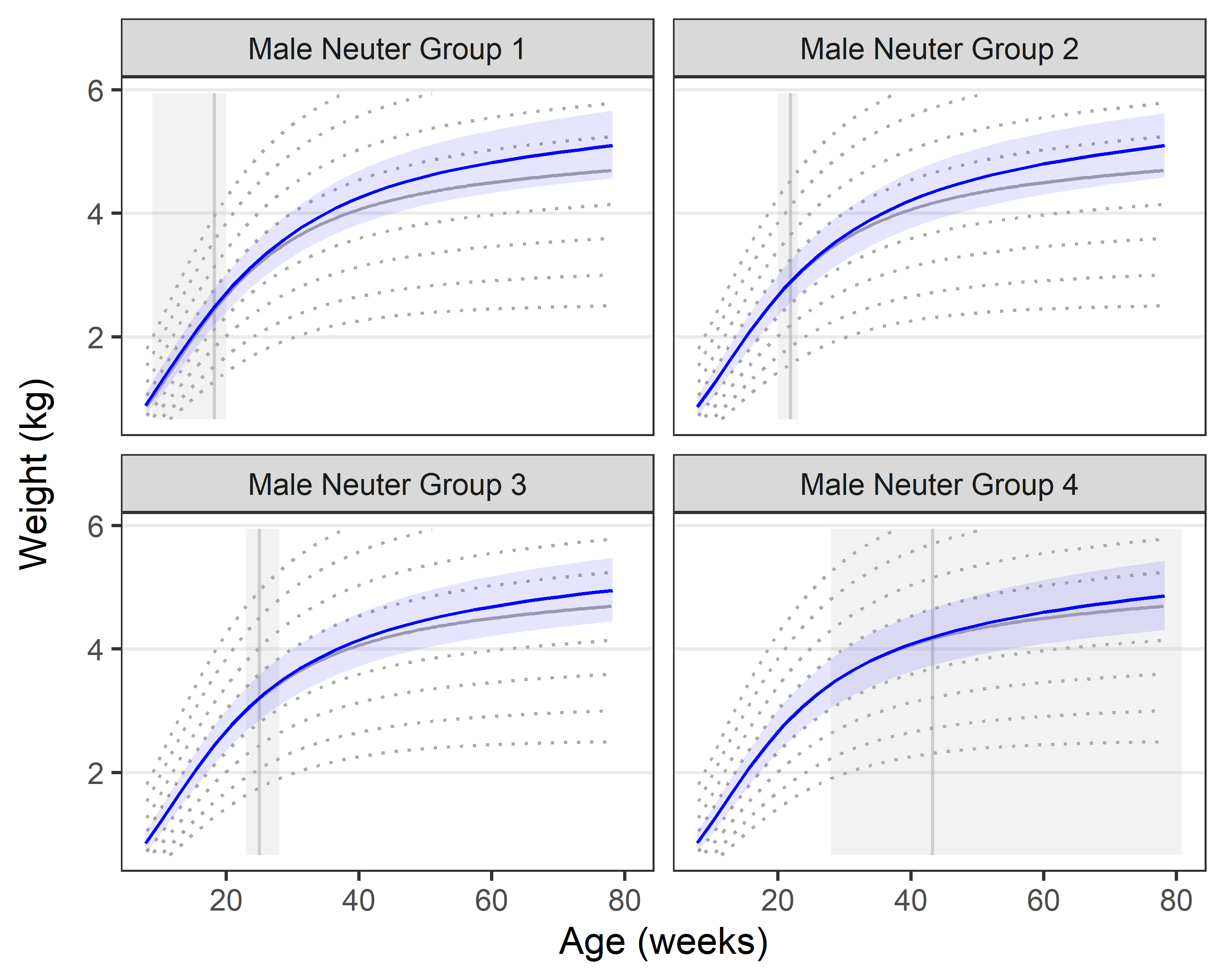

Supplement: S1 Fig — Neuter Groups 1–4 represent, respectively, neutering ages of up to 20 weeks (0.7k cats), 20–23 weeks (2.1k cats), 23–28 weeks (2.5k cats) and >28 weeks (3.0k cats). Groups calculated from the lower quartile, median and upper quartile of ages at all neutering procedures performed on DSH cats between April 1994 and November 2016. The solid blue line represents the mean trajectory, whilst the blue-shaded ribbon represents the interquartile range. The grey shaded area represents the neutering age range for the group, the solid grey vertical line shows the median observed neutering age and the dashed lines represent the standard growth centiles. In all groups, there was an upwards inclination in growth trajectory relative to the standards, which was most marked in neuter groups 1 and 2. (TIF) [file pone.0283016.s001.tif]

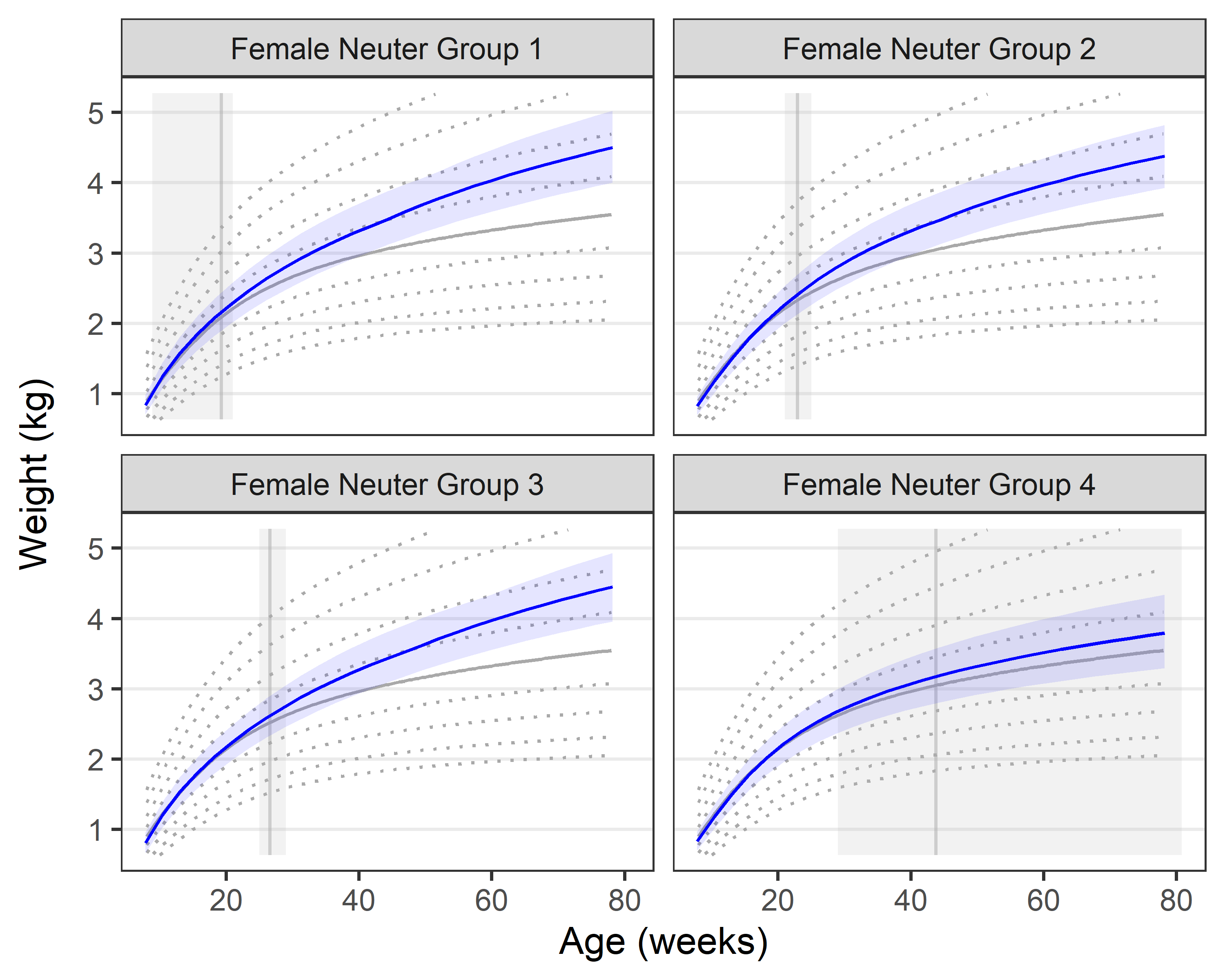

Supplement: S2 Fig — Neuter Groups 1–4 represent, respectively, neutering ages of up to 21 weeks (1.0k cats), 21–25 weeks (2.7k cats), 25–29 weeks (3.0k cats) and >29 weeks (3.8k cats). Groups calculated from the lower quartile, median and upper quartile of ages at all neutering procedures performed on DSH cats between April 1994 and November 2016. The solid blue line represents the mean trajectory, whilst the blue-shaded ribbon represents the interquartile range. The grey shaded area represents the neutering age range for the group, the solid grey vertical line shows the median observed neutering age and the dashed lines represent the standard growth centiles. In all groups, there was an upwards inclination in growth trajectory relative to the standards, which was most marked in neuter groups 1 and 2. (TIF) [file pone.0283016.s002.tif]

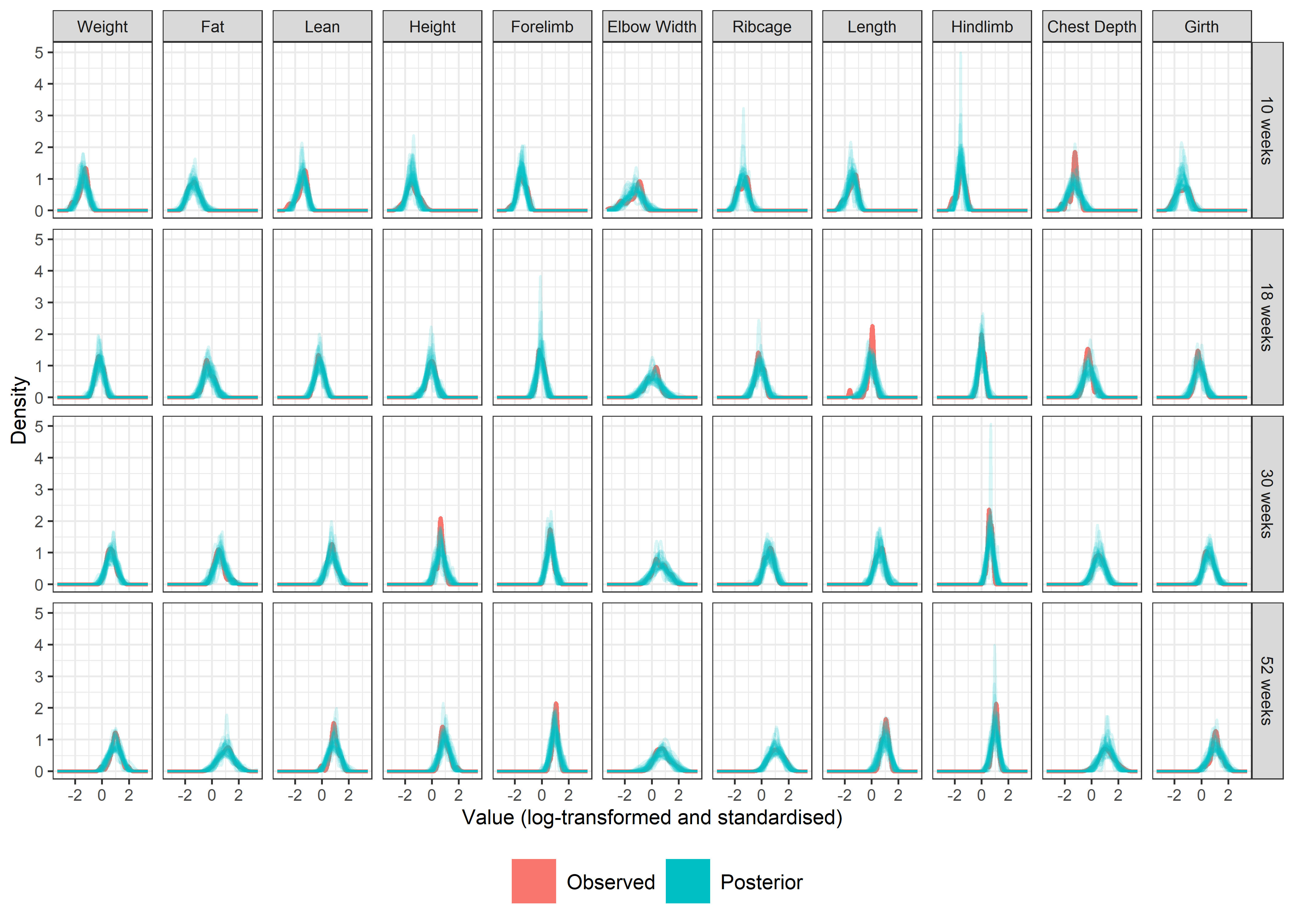

Supplement: S3 Fig — Variables are shown on the scale used for modelling, i.e., log-transformed and standardised with the z-transformation. For most measurements, posterior densities corresponded well with the respective observed densities. The main exceptions were chest depth and elbow width, at 10wk, and length at 18wk. Height at 30wk and forelimb at 52 weeks also showed some deviation, albeit smaller. (TIF) [file pone.0283016.s003.tif]
