# Supplementary material for: Comparison of growth in neutered Domestic Shorthair kittens with growth in sexually-intact cats
Source: PLoS One. 2023 Mar 15;18(3):e0283016. doi: 10.1371/journal.pone.0283016 (PMC10016642; doi:10.1371/journal.pone.0283016)
Supplement: S1 Table — (PDF) [file pone.0283016.s004.pdf]

Measure	Units	Week	Estimated Mean and 95% credible Interval		Fold Change vs 10wks		
			Sexually Intact	Neutered	Sexually Intact	Neutered	Difference
Weight	kg	10	1.08 (0.919, 1.29)	1.03 (0.876, 1.20)			
		18	2.02 (1.71, 2.41)	1.91 (1.64, 2.26)	1.87 (1.57, 2.21)	1.86 (1.58, 2.2)	0.996 (0.786, 1.27)
		30	2.88 (2.49, 3.37)	3.34 (2.85, 3.87)	2.66 (2.22, 3.10)	3.24 (2.72, 3.86)	1.22 (0.954, 1.60)
		52	3.18 (2.72, 3.75)	4.06 (3.44, 4.84)	2.94 (2.42, 3.38)	3.94 (3.34, 4.71)	1.34 (1.07, 1.72)
Fat	kg	10	0.150 (0.106, 0.206)	0.130 (0.0941, 0.184)			
		18	0.377 (0.267, 0.522)	0.318 (0.234, 0.472)	2.52 (1.84, 3.62)	2.44 (1.65, 3.39)	0.971 (0.608, 1.52)
		30	0.640 (0.461, 0.930)	0.723 (0.518, 1.02)	4.27 (2.87, 6.39)	5.55 (3.79, 7.69)	1.3 (0.771, 2.15)
		52	0.739 (0.536, 1.02)	1.23 (0.886, 1.70)	4.93 (3.66, 7.11)	9.42 (6.78, 13.0)	1.91 (1.09, 3.21)
Lean	kg	10	0.921 (0.800, 1.06)	0.873 (0.750, 0.988)			
		18	1.60 (1.39, 1.81)	1.55 (1.34, 1.79)	1.73 (1.48, 1.99)	1.77 (1.52, 2.03)	1.02 (0.812, 1.23)
		30	2.15 (1.90, 2.47)	2.51 (2.18, 2.88)	2.34 (2.00, 2.69)	2.87 (2.51, 3.39)	1.23 (0.982, 1.48)
		52	2.31 (2.01, 2.66)	2.7 (2.34, 3.10)	2.51 (2.19, 2.87)	3.09 (2.71, 3.52)	1.23 (1.03, 1.48)
Height	cm	10	15.9 (14.6, 17.1)	16.4 (15.3, 17.6)			
		18	21.1 (19.5, 22.7)	20.4 (19.0, 21.6)	1.32 (1.23, 1.44)	1.24 (1.15, 1.35)	0.937 (0.85, 1.04)
		30	23.3 (21.5, 24.9)	24.1 (22.4, 25.9)	1.46 (1.35, 1.57)	1.47 (1.36, 1.59)	1.00 (0.892, 1.11)
		52	24.6 (22.9, 26.5)	25.7 (23.9, 27.5)	1.55 (1.44, 1.69)	1.56 (1.44, 1.69)	1.01 (0.896, 1.13)
Forelimb	cm	10	7.18 (6.75, 7.63)	7.09 (6.70, 7.52)			
		18	9.31 (8.84, 9.84)	9.31 (8.83, 9.84)	1.30 (1.20, 1.40)	1.31 (1.22, 1.40)	1.01 (0.922, 1.13)

Measure	Units	Week	Estimated Mean and 95% credible Interval		Fold Change vs 10wks		
			Sexually Intact	Neutered	Sexually Intact	Neutered	Difference
		30	10.6 (9.87, 11.2)	10.8 (10.2, 11.4)	1.47 (1.38, 1.60)	1.52 (1.41, 1.63)	1.03 (0.932, 1.13)
		52	11.1 (10.5, 11.9)	11.6 (10.9, 12.2)	1.55 (1.45, 1.68)	1.63 (1.51, 1.74)	1.05 (0.955, 1.18)
Elbow Width	cm	10	1.64 (1.55, 1.72)	1.62 (1.54, 1.71)			
		18	1.87 (1.78, 1.98)	1.83 (1.74, 1.93)	1.14 (1.09, 1.22)	1.13 (1.07, 1.20)	0.987 (0.917, 1.07)
		30	1.93 (1.83, 2.04)	1.96 (1.87, 2.07)	1.18 (1.11, 1.25)	1.21 (1.14, 1.28)	1.02 (0.949, 1.10)
		52	1.95 (1.85, 2.06)	2.00 (1.90, 2.11)	1.19 (1.13, 1.27)	1.23 (1.16, 1.31)	1.03 (0.948, 1.13)
Ribcage	cm	10	20.6 (18.9, 22.3)	20.1 (18.6, 21.7)			
		18	27.9 (25.7, 30.6)	26.9 (24.7, 28.9)	1.35 (1.23, 1.48)	1.34 (1.22, 1.48)	0.990 (0.858, 1.13)
		30	31.8 (29.0, 34.6)	33.4 (30.9, 36.0)	1.54 (1.40, 1.67)	1.66 (1.51, 1.83)	1.08 (0.933, 1.22)
		52	34.2 (31.6, 36.9)	39.4 (36.3, 42.5)	1.65 (1.51, 1.81)	1.96 (1.77, 2.15)	1.18 (1.02, 1.36)
Length	cm	10	23.7 (22.2, 25.8)	23.4 (21.8, 25.2)			
		18	31.8 (29.7, 34.0)	30.7 (28.2, 32.8)	1.34 (1.24, 1.44)	1.31 (1.23, 1.43)	0.981 (0.886, 1.09)
		30	35.2 (32.7, 37.7)	36.5 (33.9, 38.9)	1.48 (1.38, 1.59)	1.56 (1.45, 1.68)	1.05 (0.956, 1.16)
		52	38.0 (35.5, 40.6)	39.3 (36.7, 42.0)	1.60 (1.49, 1.73)	1.68 (1.56, 1.80)	1.05 (0.939, 1.15)
Hindlimb	cm	10	8.83 (8.42, 9.22)	8.68 (8.25, 9.05)			
		18	11.6 (11.1, 12.1)	11.6 (11.1, 12.2)	1.31 (1.25, 1.38)	1.34 (1.26, 1.41)	1.02 (0.94, 1.10)
		30	12.9 (12.4, 13.5)	13.1 (12.6, 13.7)	1.46 (1.38, 1.54)	1.51 (1.43, 1.60)	1.04 (0.964, 1.12)
		52	13.5 (12.9, 14.1)	14.2 (13.6, 14.9)	1.53 (1.46, 1.62)	1.64 (1.56, 1.72)	1.07 (0.99, 1.16)

Measure	Units	Week	Estimated Mean and 95% credible Interval		Fold Change vs 10wks		
			Sexually Intact	Neutered	Sexually Intact	Neutered	Difference
Chest Depth	cm	10	7.25 (6.57, 7.92)	6.95 (6.30, 7.59)			
		18	9.11 (8.29, 10.1)	9.16 (8.37, 10.0)	1.26 (1.10, 1.39)	1.32 (1.19, 1.46)	1.05 (0.895, 1.24)
		30	10.9 (9.94, 11.8)	11.1 (10.0, 12.3)	1.50 (1.35, 1.69)	1.59 (1.41, 1.77)	1.06 (0.904, 1.23)
		52	11.9 (10.8, 13.2)	13.2 (12.0, 14.6)	1.64 (1.47, 1.82)	1.90 (1.7, 2.13)	1.16 (0.966, 1.38)
Girth	cm	10	20.1 (18.3, 22.0)	19.1 (17.3, 21.0)			
		18	27.4 (24.9, 30.1)	26.8 (24.4, 29.5)	1.36 (1.23, 1.53)	1.40 (1.27, 1.56)	1.03 (0.870, 1.21)
		30	32.2 (29.4, 35.2)	33.3 (30.5, 37.6)	1.60 (1.44, 1.79)	1.74 (1.54, 1.94)	1.09 (0.909, 1.25)
		52	34.9 (31.5, 38.1)	39.7 (36.0, 43.5)	1.74 (1.56, 1.93)	2.08 (1.85, 2.32)	1.20 (1.04, 1.39)

Bodyweight was measured by electronic scales; fat mass and lean mass calculated by dual-energy X-ray absorptiometry (Hologic QDR-1000 W; Hologic, Inc., Waltham, MA, USA); Height was measured at the points of the scapula; forelimb bone measured from olecranon to carpus; elbow width, measured across the humeral condyles; ribcage measured at the deepest part of the thorax; length measured from manubrium of the sternum to a parallel point below the anus; hindlimb bone measured from patella to tarsus; chest depth measured mid-thorax from spine to the deepest part; girth measured around the narrowest point of the waist. All measurements are shown to 3 significant figures.
